# Mapping the ischemic penumbra and predicting stroke progression in acute ischemic stroke: the overlooked role of susceptibility weighted imaging

**DOI:** 10.1186/s13244-019-0810-y

**Published:** 2020-01-13

**Authors:** Eman A. F. Darwish, Maha Abdelhameed-El-Nouby, Eman Geneidy

**Affiliations:** Department of Radiology, Faculty of Medicine, Ain Shams University, Abbassiya, Cairo, 11566 Egypt

**Keywords:** Susceptibility weighted imaging, Asymmetrically prominent veins, DWI-SWI mismatch, Penumbra, Stroke

## Abstract

**Objectives:**

Asymmetrically prominent veins (APVs) detected on susceptibility weighted imaging (SWI) in acute stroke patients are assumed to signify compromised cerebral perfusion. We aimed to explore the role of APVs in identifying the ischemic penumbra and predicting stroke progression in acute stroke patients

**Methods:**

Twenty patients with a middle cerebral artery ischemic infarction presenting within 24 h of symptoms onset underwent SWI following our standard MR stroke protocol imaging sequences which included diffusion-weighted imaging (DWI). Follow-up (FUP) FLAIR images were obtained at least 5 days after the initial MRI study. The Alberta Stroke Program Early CT Score (ASPECTS) was used to determine the initial infarct size, extent of APVs and final infarct size on initial DWI, SWI, and FUP images respectively. For each patient, SWI was compared with DWI images to determine match/mismatch of their respective ASPECTS values and calculate mismatch scores, whereas acute DWI findings were compared with follow-up images to identify infarct growth (IG) and calculate infarction growth scores (IGS).

**Results:**

IG occurred in 6/10 patients with a positive DWI-SWI mismatch and in none of the patients without a positive DWI-SWI mismatch. A positive DWI/SWI mismatch was significantly associated with IG (*χ*^*2*^ = 8.57, *p* = 0.0138, Cramer’s *V* = 0.65). A significant inverse correlation was found between SWI ASPECTS and IGS (*r*_s_ = − 0.702, *p* = 0.001). DWI-SWI mismatch scores were strongly correlated with IGS. (*r*_s_ = 0.788, *p* = 0.000)

**Conclusion:**

A positive DWI-SWI mismatch is an indicator of the ischemic penumbra and a predictor of infarct expansion if left untreated.

## Keypoints

- Asymmetrically prominent veins are a sign of reduced cerebral perfusion
- A positive DWI-SWI mismatch is a sign of salvageable brain tissue
- A positive DWI-SWI mismatch is a predictor of an overall increase in infarct size.
- Cases with no or negative mismatch should be carefully scrutinized for a hidden mismatch

## Introduction

Arterial ischemic stroke (AIS) represents about 80% of all strokes and is one of the leading causes of mortality and morbidity worldwide. The middle cerebral artery (MCA) territory is the most frequent vascular territory to be involved in an ischemic incident due to its large size and the direct flow of blood from the internal carotid artery (ICA) into the MCA, presenting a direct path for thromboembolism [1].

Restoration of blood flow via intravenous thrombolysis (IVT) or endovascular treatment (EVT) is currently the only functional treatment for AIS. Central to this timely intervention is the concept that there exists a considerable amount of tissue in danger of progression to irreversibly infarcted tissue, which could be salvaged if rapid reperfusion is established within a suitable time frame; the so-called ischemic penumbra [2]. Detection of the ischemic penumbra is thus crucial for starting re-canalization therapy and for predicting stroke progression and deterioration in patients with AIS [3]. For a long time, computed tomography perfusion (CTP) has been considered as the most suitable option for imaging of acute stroke patients due to the widespread availability of CT machines, short scanning times, and the relatively low examination cost. However, radiation exposure and the use of iodinated contrast media which is contraindicated in patients with history of an anaphylactic reaction and renal impairment, along with the improved magnetic resonance imaging speed has led to increased utilization of MRI-based sequences including diffusion-weighted imaging (DWI) and dynamic susceptibility contrast-enhanced (DSC) perfusion-weighted imaging (PWI) [4]. Although challenged as being a perfect representative of the ischemic penumbra, a mismatch between PWI and DWI, where the penumbra is defined as an area of critical hypo-perfusion without corresponding diffusion restriction, is currently the universally accepted strategy to identify the ischemic penumbra, foretell infarct growth, and discern patients who are most likely to benefit from re-canalization therapies [5]. Nevertheless, there are certain difficulties hindering the incorporation of DSC-PWI into the routine workup of patients with AIS including the need for well-trained medical or technical personnel to perform the required post-processing, and the necessary administration of an intravenous gadolinium-based contrast agent (GBCA) which is risky in patients with severe renal impairment denoted by glomerular filtration rate (GFR) levels below 30 ml/min. This is of particular importance in countries where renal failure is prevalent [6]. Additionally, with increased reports addressing the safety of GBCAs due to their recently discovered deposition in many organs including the brain, the need for a contrast free imaging technique is on the rise [7]. Arterial spin labeling (ASL) perfusion has been introduced as an alternative tool for assessing cerebral blood flow without the need for contrast administration, but currently it is limited to research purposes and is not widely used in mainstream clinical practice [6].

Susceptibility-weighted imaging (SWI) is a readily available, high-spatial-resolution, three-dimensional, gradient-echo T2* MR technique characterized by accentuated sensitivity for paramagnetic substances, such as deoxyhemoglobin, iron, and calcifications. The physiological compensatory response that comes into play shortly following a reduction in cerebral perfusion pressure (CPP) secondary to cerebral vascular occlusion prompts an increase in oxygen extraction fraction (OEF) in an effort to keep cerebral metabolic rate of oxygen (CMRO_2_) as close to normal as possible [2, 8]. This increase in OEF leads to an increase in the ratio of deoxyhemoglobin to oxyhemoglobin in the veins that drain areas of defective perfusion. Given the sensitivity of SWI to the paramagnetic properties of deoxyhemoglobin, the relative increase in the level of deoxyhemoglobin in the veins draining ischemic tissue leads to greater conspicuity of those veins in terms of increased hypointensity, increased number or increased caliber as compared to the veins draining the contralateral normal side. Thus, by focusing on the venous drainage, SWI can indirectly delineate hypoperfused brain areas, and it has been postulated that a mismatch between the size of the infarcted core on DWI and the extent of the asymmetrically prominent veins (APVs) seen on SWI represents ischemic tissue which is at risk of becoming infarcted if adequate perfusion is not restored in time [5, 6, 9–12].

The aims of this study were to evaluate the ability of DWI/SWI mismatch to identify potentially salvageable brain tissue (penumbra) and to predict infarct growth.

## Methods

### Patients

Adult patients clinically suspected of having an acute MCA territorial infarction, presenting within no more than 24 h of symptom onset were referred to our department by the attending neurologist in the emergency unit. All patients underwent an MRI protocol tailored specifically for stroke patients which included T1, T2, and diffusion-weighted sequences, fluid-attenuated inversion recovery (FLAIR). Only those patients confirmed as having an acute, non-lacunar, ischemic infarction of the MCA territory were enrolled in the study and underwent further evaluation by susceptibility-weighted imaging.

Excluded from this study were patients with the hemorrhagic transformation of ischemic stroke evident on initial MR images. (N.B. Patients who developed subsequent hemorrhagic changes after initial MR imaging were not excluded from the study). Agitated patients that were unable to remain still, or patients with a poor general condition who were unable to tolerate the relatively lengthy period of time it took to perform SWI, and patients who received thrombolytic therapy or underwent endovascular therapeutic procedures were excluded.

Follow-up FLAIR images were obtained not less than 5 days after the initial MRI for evaluation of the final infarction size. Patients who did not come in for their follow-up examination were excluded from the study.

Informed consent was obtained from all patients and this study was approved by our institutional review board and fulfilled ethical standards.

### Image acquisition

Magnetic resonance imaging studies were performed with a 1.5-T MR system (Achieva, Philips). All patients underwent examination by our MRI protocol tailored specifically for stroke patients, which included the following fast imaging sequences: axial fast field echo (FFE) T1, axial turbo spin-echo (TSE) T2, FLAIR, and DWI, in addition to the SWI sequence. The various parameters for the basic MR sequences are listed in Table 1.

**Table 1:** Imaging parameters of the basic MR sequences

Sequences	Imaging plane	TR/TE (ms)	Acquisition time (s)	Voxel size (mm)	FOV (mm)	Matrix	Slice thickness (mm)	FA/BW (deg)/(Hz)
FFE T1	Axial	157/1.77	20	0.9/1.12/5	220 × 190	244 × 169	5	80/127.6
TSE T2	Axial	4845/110	38	0.9/1.12/5	220 × 188	256 × 147	5	90/212.9
FLAIR	Axial	6000/120	54	0.9/1.28/5	220 × 190	244 × 127	5	100/359.5

*BW* bandwidth, *deg* degrees*, FA* flip angle, *FLAIR* fluid-attenuated inversion recovery, *FFE* fast field echo, *FOV* field of view, *TE* echo time, *TR* repetition time, *TSE* turbo spin-echo

For the DWI sequence, we used single-shot spin-echo echo-planar imaging with the following parameters: TR/TE = 4124/118 ms, *b* values = 0–1000 s/mm^2^, voxel size = 1.5 × 2.21 × 5 mm, slice thickness = 5 mm, FA = 90°, BW = 17.7 Hz, FOV = 190 × 232 × 143 mm, matrix = 128 × 105 and acquisition time = 1 min, 26 s. ADC maps were automatically generated.

The SWI sequence was obtained with the following parameters: TR = 34 ms; TE = 24 ms; flip angle = 10°, bandwidth = 128.2 Hz, slice thickness = 0.5 mm with 240 slices per slab, voxel size = 1.1 × 1.1 × 0.5 mm, FOV = 230 × 190 × 120 mm, and matrix size = 208 × 173. The acquisition time was 7 min, 57 s. During post-processing, minimum intensity projection (minIP) images were reconstructed with an effective minIP thickness of 10 mm.

Follow-up FLAIR images were obtained with the same parameters described above.

### Image analysis

Analysis of the images was undertaken by two experienced neuroradiologists and decisions were reached by consensus. Acute infarction was identified in initial DW images as an area of bright signal intensity which appeared dark on the corresponding ADC map. In the follow-up FLAIR images, infarcted tissue was identified as hyperintense areas of gliosis or hypointense areas of encephalomalacia surrounded by a gliotic rim. APVs were identified in SW images when prominent cortical veins were seen on the side of the infarction relative to the contralateral normal side due to an increase in their number, caliber, or the degree of their hypointensity. The Alberta Stroke Program Early CT Score (ASPECTS) was used to assess the extent of the abnormalities described above in the DW, SW, and follow-up FLAIR images.

According to the ASPECTS scoring system, the MCA territory is divided into ten zones; caudate nucleus, lentiform nucleus, internal capsule, insula, M1, M2, and M3 (anterior, middle, and posterior third of the lower MCA territory, respectively), and M4, M5, and M6 (anterior, middle, and posterior third of the higher MCA territory, respectively). To calculate the ASPECTS score in the initial DW, SW, and follow-up FLAIR images for each patient, 1 point was deducted from 10 for each area of restricted diffusion, APVs, and hyperintensity in the DW, SW and FLAIR images respectively.

Patients were classified into one of three groups, namely infarction growth (IG) or no infarction growth (NIG) or reversal of infarction (RI), depending on the relationship between the DWI ASPECTS and the follow-up (FUP) ASPECTS determined on the follow-up FLAIR images. IG was determined when the DWI ASPECTS score was greater than the FUP ASPECTS score (DWI ASPECTS > FUP ASPECTS). NIG was determined when the ASPECTS score was equal on both DW and follow-up FLAIR images. RI was determined when the FUP ASPECTS was greater than DWI ASPECTS (DWI ASPECTS < FUP ASPECTS). The infarct growth score for each patient was calculated by subtracting FUP ASPECTS score from the DWI ASPECTS score (DWI ASPECTS-FUP ASPECTS). For every patient, the relationship between DWI ASPECTS and SWI ASPECTS was determined as follows:

- A positive mismatch was identified when the DWI ASPECTS was greater than SWI ASPECTS (the number of affected vascular territories was higher on SWI than DWI).
- A negative mismatch was identified when the DWI ASPECTS was less than the SWI ASPECTS (the number of affected vascular territories was higher on DWI than SWI).
- No mismatch was identified when the ASPECTS score was equal on both DWI and SWI.

For each patient, the DWI ASPECTS/SWI ASPECTS mismatch score was calculated by subtracting the SWI ASPECTS score from the DWI ASPECTS score.

### Statistical analysis

Non-parametric tests were used because the data were not normally distributed. Data were expressed as median and percentiles for quantitative non-parametric variables in addition to both numbers and percentage for categorized data.

Comparison of the various ordinal variables between the different patient groups was performed using the Wilcoxon rank-sum test while the chi-squared test was used for comparison of categorized data. The chi-squared test and Cramer *V* coefficient were used to investigate the association between DWI/SWI mismatch and infarction growth. To detect statistical differences between ASPECTS values of the various imaging sequences, infarct growth and mismatch scores within the same group, the Wilcoxon signed-rank test was used. The correlations between the ASPECTS scores of the various sequences were determined by using Spearman’s rank correlation tests. A statistical software package (IBM SPSS statistics, version 25.0, IBM Corp., USA, 2017–2018) was used for data analysis. Results were considered significant if the *p* value was < 0.05 and highly significant if the *p* value was < 0.01 or < 0.001.

## Results

Out of a total of 20 patients, 6 patients (30%) showed infarct growth while 14 (70%) patients did not demonstrate infarct growth. Reversal of initial DWI lesions was not encountered in any of the patients enrolled in this study. Eleven (55%) and nine (45%) patients had infarctions of the right and left MCA territories respectively. The initial MR imaging was performed at a median of 13.5 h (interquartile range 8–19.875 h) after the onset of symptoms. FUP studies were obtained at a median of 7 days (interquartile range 5–10 days) after the initial MRI.

**Fig. 1:**
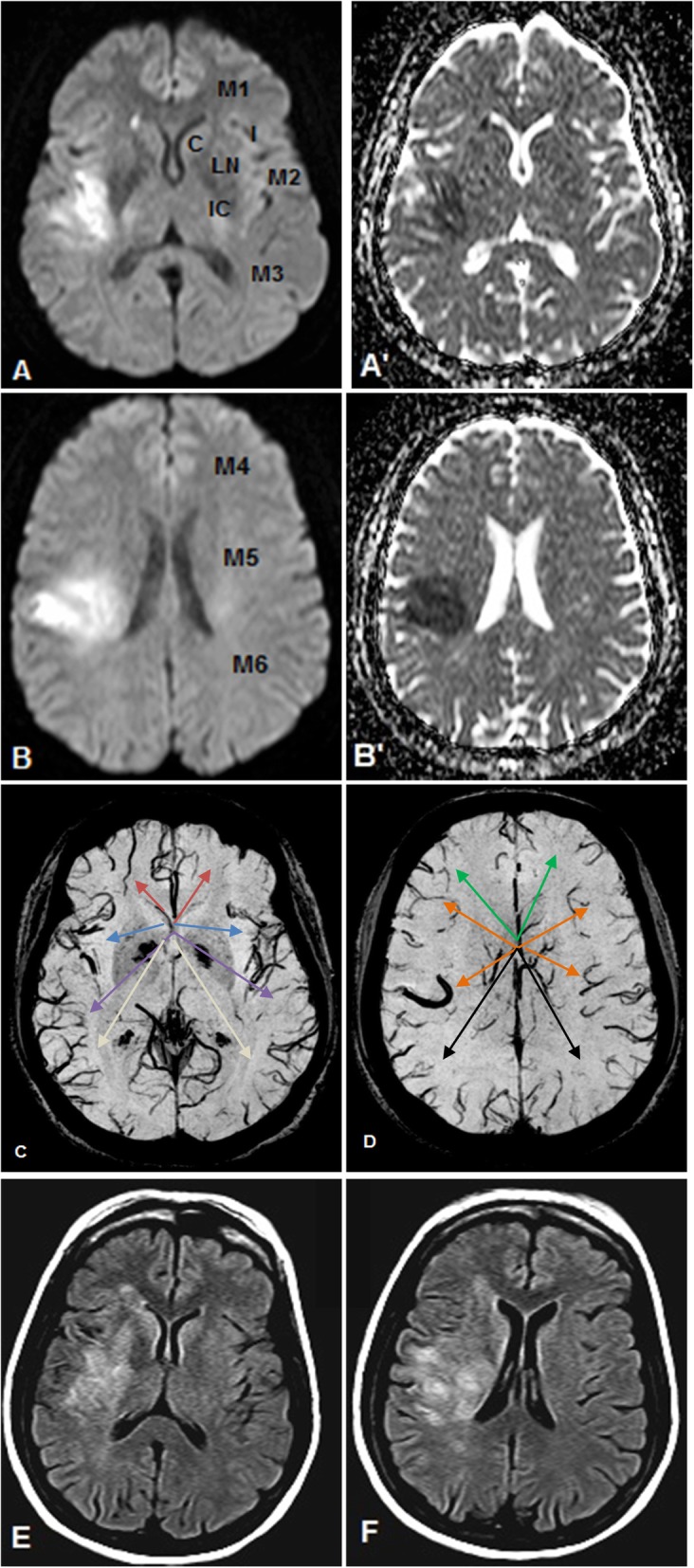
**a**–**f** 60-year-old female with right MCA territory infarct. The 10 MCA zones designated by the ASPECTS scoring system are *C* caudate, *L* lentiform nucleus, *IC* internal capsule, *I* insular ribbon, *M1–6* cortical regions (M1–3 at the level of basal ganglia, M4–6 at the level cranial to the basal ganglia). Initial DW images and their corresponding ADC maps at the basal ganglia level (**a**, **a**') and the supraganglionic level (**b**, **b**') reveal the presence of acute infarction in I, M1, M2, and M5 regions with a resultant ASPECTS score of 6. SW images at the basal ganglia level (**c**) and the supraganglionic level (**d**) reveal the presence of APVs, in terms of either density and/or caliber and/or hypointensity, in I (blue arrow) and in M1–6 regions (red, purple, gray, green, orange, and black arrows respectively) with a resultant ASPECTS score of 3 (similar colored arrows point to the corresponding areas in the contralateral normal hemisphere for comparison). DWI/SWI mismatch score is 3. Follow up FLAIR images, obtained 10 days later, at the basal ganglia level (**e**) and the supraganglionic level (**f**) reveal in addition to the originally infarcted zones, infarction growth in L, IC, and M4, giving an overall ASPECTS score of 3 and an IGS of 3. The faint hyperintensities noted at M3 and M6 zones were considered to be ischemic foci and not newly infarcted areas as they were evident in the FLAIR images of the initial MRI with no corresponding diffusion restriction

**Fig. 2:**
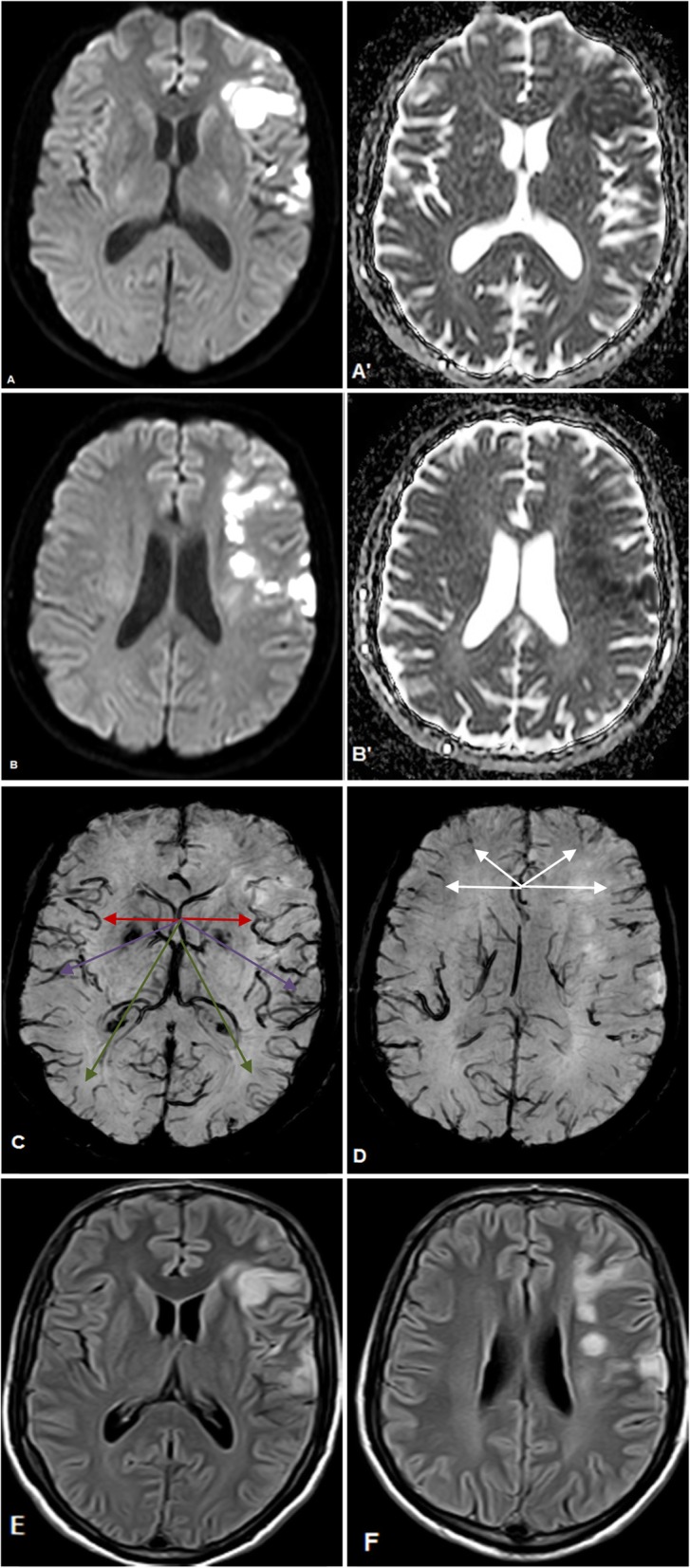
**a**–**f** 52-years-old male with left MCA territory infarct. Initial DW images and their corresponding ADC maps at the basal ganglia level (**a**, **a**') and the supraganglionic level (**b**, **b**') reveal the presence of an acute infarction in I, M1, M2, M4 and M5 regions with a resultant ASPECTS score of 5. SW images at the basal ganglia level (**c**) and the supraganglionic level (**d**) reveal the presence of APVs, in terms of either density and/or caliber and/or hypointensity, in I (red arrow) and in M2, M3 and to a milder extent in M4 regions (purple, green, and white arrows respectively) with a resultant ASPECTS score of 6 (similar colored arrows point to the corresponding areas in the contralateral normal hemisphere for comparison). DWI/SWI mismatch score is − 1. Despite the overall negative mismatch, the presence of APVs in the M3 zone, which did not show an acute infarction in the initial DW images, indicates the presence of a hidden mismatch. However, follow up FLAIR images obtained 5 days later, at the basal ganglia level (**e**) and the supraganglionic level (**f**), reveal the presence of signal alteration in precisely the same zones that showed restriction in the initial DW images with no infarct progression

Among the NIG group, only 4/14 (28.6%) patients showed a positive mismatch, while the remaining patients showed either no mismatch (*n* = 6) or a negative mismatch (*n* = 4). On the other hand, among the IG group (*n* = 6); all (100%) of the patients displayed a positive mismatch. Examples of the different mismatch patterns are seen in Figs. 1 and 2. An association between DWI/SWI mismatch and IG was found, where a positive DWI/SWI mismatch was significantly associated with infarct growth on follow-up imaging; *χ*^*2*^ = 8.57, *p* = 0.0138, Cramer’s *V* = 0.65. DWI/SWI mismatch and IG scores were significantly higher in the IG group than the NIG group while the SWI ASPECTS score was significantly lower in the IG group than the NIG. Patients’ demographic data and imaging findings are summarized in Table 2.

**Table 2:** Patients’ demographic data and imaging findings

		NIG group	IG group	*p* value	Sig.
		No. = 14	No. = 6		
Age (years)	Median (IQR)	64 ( 52–67.75)	60 (51–69.5)	0.934	NS^*^
	Range	36–73	45–74		
Gender	Females	4 (28.6%)	4 (66.7%)	0.111	NS^**^
	Males	10 (71.4%)	2 (33.3%)		
Infarct side	Right	7 (50.0%)	4 (66.7%)	0.492	NS^**^
	Left	7 (50.0%)	2 (33.3%)		
Mismatch	SWI aspects > initial DWI aspects (negative mismatch)	4 (28.6%)	0 (0.0%)		
	SWI aspects < initial DWI aspects (positive mismatch)	4(28.6%)	6 (100%)	0.014	S^**^
	No SWI–DWI mismatch	6 (42.9%)	0 (0.0%)		
Initial DWI ASPECTS	Median(IQR)	7 (4.75–8)	7.5 (6–8)	0.356	NS^*^
	Range	1–9	6–9		
SWI aspects	Median(IQR)	7 (5–8)	3.5 (2.5–4.5)	0.004	HS^*^
	Range	4–9	1–6		
FUP ASPECTS	Median(IQR)	7 (4.75–8)	5.5 (3–6.5)	0.261	NS^*^
	Range	1–9	3–8		
DWI/SWI mismatch score	Median (IQR) Range	0 (− 1.25–2) − 6–2	3.5 (3–4.5) 3–6	0	HS^*^
Infarct growth score	Median (IQR) Range	0 (0) 0	2 (1.75–3) 1–3	0	HS^*^
Time to MRI (h)	Median (IQR) Range	14 (7.875–20.125) 6–24	12.5 (7.75–20.5) 7–22	0.869	NS^*^
Follow-up time (days)	Median (IQR)	7 (5–9.25)	8.5 (5–14)	0.498	NS^*^
	Range	5–15	5–14		

*ASPECTS* Alberta Stroke Program Early CT Score, *DWI* diffusion-weighted imaging, *FUP* follow-up, *HS* highly significant, *IG* infarct growth, IQR- interquartile range, *NIG* non-infarct growth, *NS* non-significant, *S* significant, *SWI* susceptibility-weighted imaging

^*^Wilcoxon rank-sum test

^**^Chi-squared test

Out of a total of 200 vascular zones in 20 patients, 70 vascular territories were acutely infarcted on initial DWI. The most frequently affected vascular territory was M5 (*n* = 19, 27.14%) while the least common territory to be involved by an acute infarction was caudate which was affected once only (1.43%). On SWI, 50 out of 70 (71.4%) acutely infarcted vascular territories showed APVs. Table 3 shows the distribution of APVs in the acutely infarcted MCA territories.

**Table 3:** Distribution of APVs in the acutely infarcted MCA territories in all patients

	Acute infarction	
	APV −ve	APV +ve
Caudate	0	1
LN	3	3
IC	1	1
INSULA	5	8
M1	3	3
M2	0	12
M3	2	0
M4	4	2
M5	0	19
M6	2	1

*APVs* asymmetrically prominent veins

Out of a total of 60 vascular territories in the IG group, 16 zones displayed an acute infarction on initial DWIs and were assessed separately for the presence of prominent veins as mentioned above. Thirteen out of the 44 remaining territories showed infarct growth, 7 of which showed prominent veins while 31/44 zones showed no infarction growth of which 17 showed prominent veins. Out of a total of 140 vascular territories in the NIG group, 54 were acutely infarcted. Out of the remaining 86 regions, none of which showed infarct growth, only 13 zones displayed asymmetrically prominent veins. Tables 4 and 5 show the distribution of APVs and the presence or absence of infarct growth in the different MCA territories in both IG and NIG groups respectively.

**Table 4:** Distribution of APVs and presence or absence of infarct growth in the MCA territories in the IG group

	Infarction growth + ve		Infarction growth −ve	
	APVs −ve	APVs +ve	APVs −ve	APVs +ve
Caudate	1	0	4	0
LN	2	0	2	0
IC	3	0	2	0
INSULA	0	0	1	2
M1	0	0	3	1
M2	0	2	0	2
M3	0	0	0	6
M4	0	3	1	2
M5	0	1	0	0
M6	0	1	1	4

*APVs* asymmetrically prominent veins, *APVs −ve/+ve* negative(absent)/positive (present) asymmetrically prominent veins

**Table 5:** Distribution of APVs in the MCA territories in NIG group

	Infarction growth –ve	
	APVs −ve	APVs +ve
Caudate	14	0
LN	9	1
IC	12	1
INSULA	4	0
M1	8	2
M2	2	2
M3	11	1
M4	8	0
M5	0	0
M6	9	2

*APVs* asymmetrically prominent veins, *APVs −ve/+ve* negative(absent)/positive (present) asymmetrically prominent veins

**Fig. 3:**
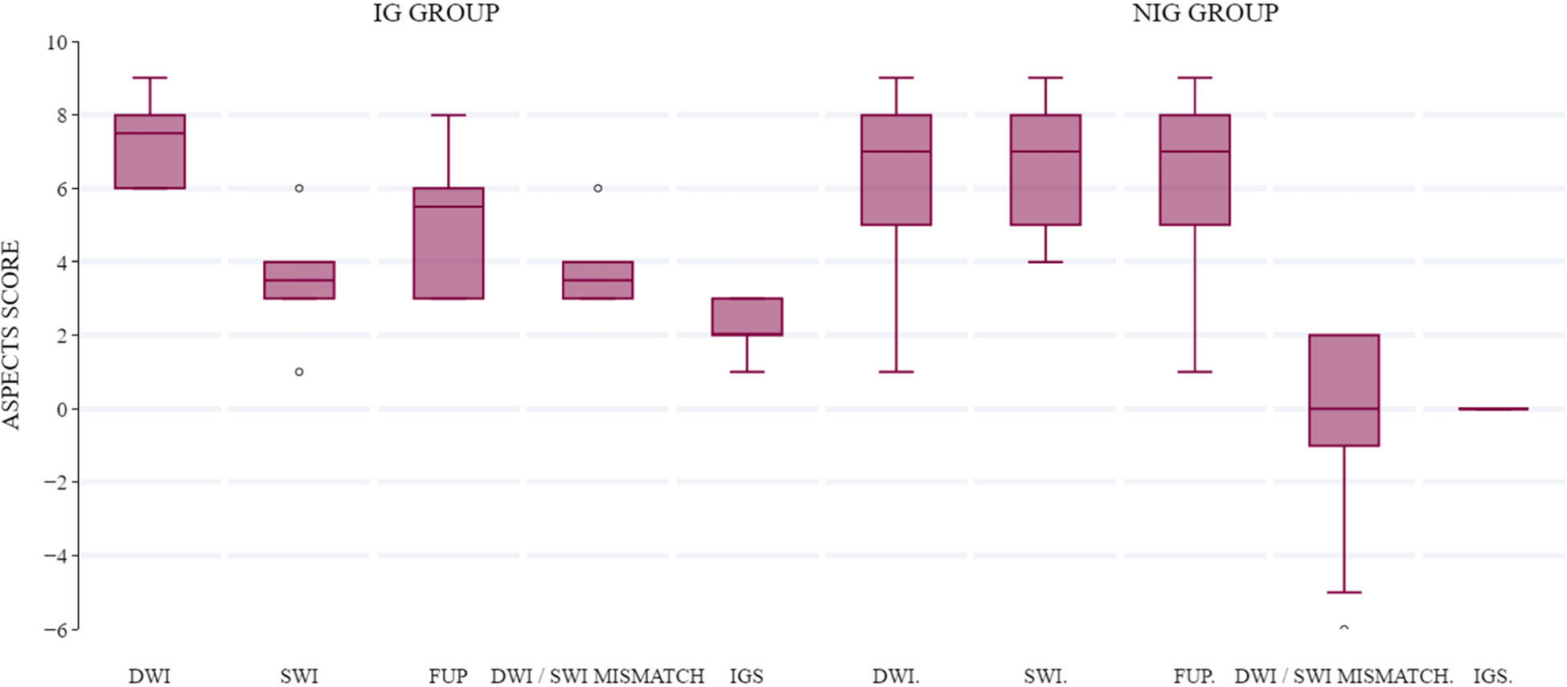
Box plots of DWI, SWI, and FUP ASPECTS values as well as DWI/SWI mismatch and IG scores in both patient groups. Middle lines represent median values, boxes represent 25th to 75th percentiles, and whiskers demonstrate range. *DWI* diffusion-weighted imaging, *SWI* susceptibility-weighted imaging, *FUP* follow up, *IG* infarction growth, *IGS* infarction growth score, *NIG* no infarction growth

Within the NIG group, no significant difference was seen between the SWI, DWI, and FUP ASPECTS scores (*p* = 0.774) or between the DWI/SWI mismatch and IG scores (*p* = 0.774), while both the DWI/SWI mismatch and IG scores were significantly lower than the SWI, DWI, and FUP ASPECTS (*p* = 0.001). Within the IG group, the SWI ASPECTS and FUP ASPECTS, DWI/SWI mismatch, and IG scores were significantly lower than the DWI ASPECTS values (*p* = 0.026, *p* = 0.026, *p* = 0.027, *p* = 0.027 respectively). No significant difference existed between SWI ASPECTS and FUP ASPECTS (*p* = 0.059), DWI/SWI mismatch score (*p* = 0.655), and IG score (*p* = 0.141), or between FUP ASPECTS and both the DWI/SWI mismatch and IG scores (*p* = 0.141, and *p* = 0.066 respectively). Likewise, no significant difference existed between the DWI/SWI mismatch and IG scores (*p* = 0.059). Figure 3 gives an overview of the various scores within each group.

**Fig. 4:**
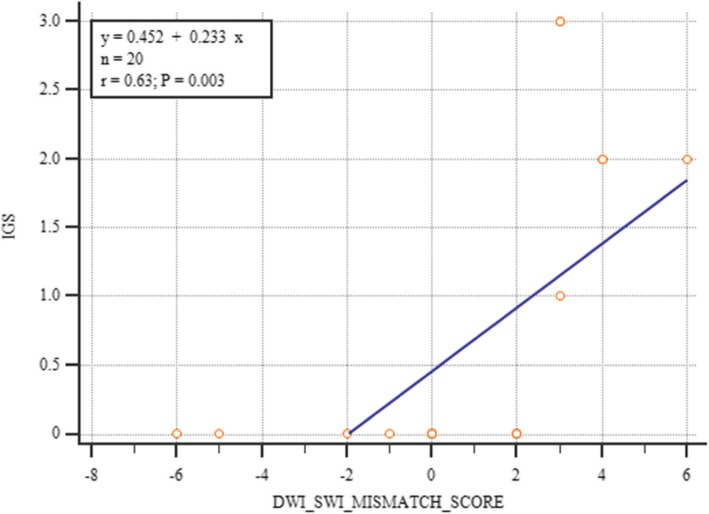
Correlation between DWI/SWI mismatch and IG scores. There was a significant positive correlation between DWI/SWI mismatch and IG scores (*n* = 20, *r*_s_ **=** 0.788, *p* = 0.000 [HS])

Table 6 displays the correlations between the various ASPECTS scores. There were significant correlations between the SWI ASPECTS and FUP ASPECTS, between SWI ASPECTS and IGS, between DWI/SWI mismatch and IG scores (Fig. 4), and between DWI ASPECTS and FUP ASPECTS.

**Table 6:** Correlations between the various scores and their statistical significance

	DWI ASPECTS		SWI ASPECTS		FUP ASPECTS		DWI/SWI mismatch score		IGS	
	*r*_s_	*p* value	*r*_s_	*p* value	*r*_s_	*p* value	*r*_s_	*p* value	*r*_s_	*p*-value
DWI ASPECTS	–	–	0.308	0.184 ( NS)	0.867	0.000 (HS)	0.390	0.089 (NS)	0.128	0.592 (NS)
SWI ASPECTS	–	–	–	–	0.555	0.011 (S)	0.748	0.000 (HS)	− 0.702	0.001 (HS)
FUP ASPECTS	–	–	–	–	–	–	0.049	0.838 (NS)	− 0.331	0.154 (NS)
DWI/SWI mismatch score	–	–	–	–	–	–	–	–	0.788	0.000 (HS)

*ASPECTS* Alberta Stroke Program Early CT Score, *DWI* diffusion weighted imaging, *FUP* follow up, *HS* highly significant, *IGS* infarct growth score, *NS* non-significant, *R*_*S*_ Spearman rank correlation coefficient, *S* significant, *SWI* susceptibility-weighted imaging

The sensitivity, specificity, positive predictive value, negative predictive value, and efficacy of a positive DWI/SWI mismatch in predicting infarction growth were 100% (95% CI = 54.07–100%), 71.43% (95% CI = 41.90–91.61%), 60% (95% CI = 39.59–77.45%), 100% (95% CI = 65.55–100%), 80% (95% CI = 56.34–94.27%) respectively.

## Discussion

The results of this study showed that a positive DWI/SWI mismatch is an indicator of salvageable brain tissue, which without intervention is at risk of stroke progression, with higher mismatch scores suggesting more extensive infarction growth. Our findings were in line with the results of several previous studies [5, 6, 10, 13–15]. Controversially, a few studies suggested that the unilaterally prominent vessels seen on SWI in stroke patients could present efficient leptomeningeal arterial collaterals or that their presence is associated with an efficient collateral flow [16, 17]. This presents an obvious contradiction to this study, as an adequate collateral flow has long since been established as an indicator of a favorable outcome following an ischemic event [9, 18–20]. This hypothesis requires further validation, however, especially in view of studies by Kesavadas et al. and Verma et al. where the authors found that these vessels failed to demonstrate any territorial preferences as would be expected of arteries and were conspicuously absent in cases with good leptomeningeal collateralization [8, 21].

In spite of the good correlation, we found between a positive DWI-SWI mismatch and an overall increase in infarct size, zonal analysis revealed that spatial correlation between the extent of APVs and infarct growth in individual vascular territories was suboptimal. While all of the superficial MCA territories, namely, M1–6 and insula, which showed infarction growth also displayed APVs, not all zones with APVs showed infarction growth on follow up images. This is not surprising as the exact fate of the penumbral zone is determined by a complicated interplay of factors including the state of collateral circulation, which may differ across the penumbra and the variable infarction threshold among different tissues [22]. Moreover, the OEF and the resultant venous hypointense signals increase not only in the penumbra but also in the surrounding zone of benign oligemia which is characterized by a normal CMRO_2_ and is generally, not considered to be at risk of progression to irreversible damage [2, 23]. As opposed to the superficial MCA zones, none of the deep MCA territories, namely, the caudate, lentiform nucleus, and IC, which showed infarction growth displayed APVs. The diminished sensitivity of APVs in predicting stroke progression in the deep MCA territories was explained in an older study by the fact that the thalamostriate vein, which drains the three deep zones, also drains the thalamus which is unaffected by an MCA occlusion, thus altering the ratio of deoxygenated to oxygenated blood and in turn, the degree of conspicuity of the vein on SWI [15]. Furthermore, susceptibility effects of the increasing mineralization of the basal ganglia region that occurs with age may mask the small tributaries draining these areas to the thalamostriate vein.

A positive DWI/SWI mismatch was seen in 4 out of the 14 patients with no IG in our study. This was consistent with the data from older studies which showed that a positive DWI/SWI mismatch, despite being linked to stroke progression, did not necessarily mean an inevitable increase in infarct size in every case [5, 10, 15]. We assumed that these patients developed improved collateral circulation which is known to lead to re-canalization even without the administration of a thrombolytic agent and subsequent stabilization of infarct volume [15, 18, 24–28].

Four patients in the NIG group displayed a negative DWI/SWI mismatch. Comparable findings were reported by Polan et al. [10]. Similar to an inverse PWI/DWI mismatch, a negative DWI/SWI mismatch is thought to occur in cases where a good collateral circulation leads to spontaneous re-canalization and restoration of blood flow resulting in normalization of the OEF and reduction in deoxyhemoglobin levels [29]. Adequate collateralization has been described by several authors as a cause of diminished venous hypointense signals on SWI [8, 12, 21, 30]. Other studies have suggested that the ensuing tissue necrosis which causes a marked drop in the metabolic demands of tissue and subsequently the OEF, as well as the developing cellular swelling and edema, are the reasons behind the reduced perception of APVs within the infarct core 12 to 24 h after stroke onset [6, 30–32]. We did not evaluate the impact of time on SWI findings in our study and thus, cannot verify these results, but we did note that all four patients with a negative DWI/SWI mismatch underwent the MRI within 17–23 h after symptom onset. However, other patients presenting within the same time frame demonstrated APVs and alternative patterns of DWI/SWI. Nevertheless, as the visibility of the venous susceptibility signals may potentially decrease with time, it may be more prudent to use PWI to identify the penumbra in patients presenting later than 12 h from the onset of symptoms. This is particularly important in view of the recent DAWN and DEFUSE-3 trials, whose results have supported widening the time window for thrombectomy from the classical 6 h to up to 24 h from onset of symptoms in patients with evidence of salvageable ischemic tissue [33, 34].

The limitations of our study include the small number of patients and the fact that neither positron emission tomography (PET) nor PWI were included in our imaging protocol which meant that our data could not be confirmed by comparison to PET or PWI. Interpretation of SWI was time-consuming and sometimes difficult due to differences in slice thickness between the DW images and minIP-SWI. Furthermore, APVs were determined by visual inspection and comparison rather than by objectively quantifying vessel number and caliber which may have given rise to investigator dependent bias even though image interpretation was performed by consensus. Despite the fact that the ASPECTS system is regarded as a dependable grading system that can accurately delineate the extent of ischemic change, several potential pitfalls exist. Firstly, the ASPECTS system does not take into account subtle areas of mismatch or infarct growth, which may occur in the pre-determined individual vascular territories. Secondly, the possibility of normalization or reduction of the venous BOLD signal within the infarcted core means that a no mismatch or even negative mismatch pattern could potentially occur in the presence of APVs extending into vascular territories unaffected on DWI resulting in the so-called hidden mismatch pattern which has been previously described by studies directed at investigating the various perfusion-diffusion mismatch patterns [22, 29]. We encountered a hidden mismatch in a single patient in this study who luckily did not demonstrate any infarct progression in the FUP images, yet this mismatch pattern presents one of the pitfalls of using the ASPECTS scoring system for analysis, as patients with a hidden mismatch are at risk of stroke progression, unlike patients with true inverse or no mismatch [22, 35]. Thus, a hidden mismatch should be carefully excluded whenever a negative or no mismatch pattern is found using the ASPECTS system. Alternatively, using the more meticulous co-registration method which entails fusing DW images with the minimum intensity projection SW images to produce co-registered DW/SW images, may allow more precise image interpretation leading to greater diagnostic accuracy [6, 22]. Finally, the relatively long acquisition time of SWI and its marked vulnerability to motion artifacts made it unsuitable for agitated and restless patients.

## Conclusion

In conclusion, results of this study reveal that a positive DWI/SWI mismatch is a viable indicator of ischemic penumbra and a prognosticator of infarct expansion and thus can potentially be used to guide early thrombolytic or endovascular treatments to prevent stroke progression. Therefore, we recommend the addition of SWI to the routine neuroimaging protocol for patients with suspected acute stroke when PWI is unavailable or unsuitable. However, as with any other imaging sequence, potential pitfalls exist and analysis of images should be performed by radiologists familiar with SWI and the various mismatch patterns to avoid possible misinterpretations.

## Data Availability

The datasets used in the current study are available from the corresponding author on request

## Abbreviations

ADC: Apparent diffusion coefficient
AIS: Arterial ischemic stroke
APVs: Asymmetrically prominent veins
ASL: Arterial spin labeling
ASPECTS: Alberta Stroke Program Early CT Score
BOLD: Blood oxygenation level-dependent
BW: Bandwidth
C: Caudate
CI: Confidence interval
CMRO_2_: Cerebral metabolic rate of oxygen
CPP: Cerebral perfusion pressure
CT: Computed tomography
CTP: Computed tomography perfusion
DSC: Dynamic susceptibility contrast-enhanced
DW: Diffusion-weighted
DWI: Diffusion-weighted imaging
EVT: Endovascular treatment
FA: Flip angle
FLAIR: Fluid attenuated inversion recovery
FOV: Field of view
FUP: Follow up
GBCA: Gadolinium-based contrast agent
GFR: Glomerular filtration rate
I: Insular ribbon
IC: Internal capsule
ICA: Internal carotid artery
IG: Infarction growth
IGS: Infarction growth score
IQR: Interquartile range
IVT: Intravenous thrombolysis
L: Lentiform nucleus
MCA: Middle cerebral artery
minIP: Minimum intensity projection
MRI: Magnetic resonance imaging
NIG: No infarction growth
NS: Non-significant
OEF: Oxygen extraction fraction
PET: Positron emission tomography
PWI: Perfusion-weighted imaging
QSM: Quantitative susceptibility mapping
RI: Reversal of infarction
SW: Susceptibility weighted
SWI: Susceptibility weighted imaging
TE: Echo time
TR: Repetition time
TSE: Turbo spin echo
